# Designer Benzodiazepines: A Review of Toxicology and Public Health Risks

**DOI:** 10.3390/ph14060560

**Published:** 2021-06-11

**Authors:** Pietro Brunetti, Raffaele Giorgetti, Adriano Tagliabracci, Marilyn A. Huestis, Francesco Paolo Busardò

**Affiliations:** 1 Department of Excellence of Biomedical Sciences and Public Health, Marche Polytechnic University of Ancona, Via Tronto 10, 60126 Ancona, Italy; pietrobrunetti40@gmail.com (P.B.); r.giorgetti@univpm.it (R.G.); a.tagliabracci@univpm.it (A.T.); fra.busardo@libero.it (F.P.B.); 2 Institute of Emerging Health Professions, Thomas Jefferson University, 1020 Walnut St., Philadelphia, PA 19144, USA

**Keywords:** benzodiazepine, designer, NPS, intoxication, poisoning, impairment, death

## Abstract

The rising use of designer benzodiazepines (DBZD) is a cat-and-mouse game between organized crime and law enforcement. Non-prohibited benzodiazepines are introduced onto the global drug market and scheduled as rapidly as possible by international authorities. In response, DBZD are continuously modified to avoid legal sanctions and drug seizures and generally to increase the abuse potential of the DBZD. This results in an unpredictable fluctuation between the appearance and disappearance of DBZD in the illicit market. Thirty-one DBZD were considered for review after consulting the international early warning database, but only 3-hydroxyphenazepam, adinazolam, clonazolam, etizolam, deschloroetizolam, diclazepam, flualprazolam, flubromazepam, flubromazolam, meclonazepam, phenazepam and pyrazolam had sufficient data to contribute to this scoping review. A total of 49 reports describing 1 drug offense, 2 self-administration studies, 3 outpatient department admissions, 44 emergency department (ED) admissions, 63 driving under the influence of drugs (DUID) and 141 deaths reported between 2008 and 2021 are included in this study. Etizolam, flualprazolam flubromazolam and phenazepam were implicated in the majority of adverse-events, drug offenses and deaths. However, due to a general lack of knowledge of DBZD pharmacokinetics and toxicity, and due to a lack of validated analytical methods, total cases are much likely higher. Between 2019 and April 2020, DBZD were identified in 48% and 83% of postmortem and DUID cases reported to the UNODC, respectively, with flualprazolam, flubromazolam and etizolam as the most frequently detected substances. DBZD toxicology, public health risks and adverse events are reported.

## 1. Introduction

Benzodiazepines (BZD), important forensic and clinical toxicology drugs, are widely prescribed for neurological and psychiatric disorders and are also highly abused [1,2,3]. Discovered in the mid-1950s, BZD were designed as pharmacotherapies for anxiety, panic attacks, sleep disorders and epilepsy, and they have been used as myorelaxants during surgical and orthopedic procedures [4,5]. BZD are positive allosteric modulators that enhance the binding affinity of the inotropic γ-aminobutyric acid-A receptor (GABA_A_) for GABA, the major central nervous system (CNS) inhibitory neurotransmitter [6,7]. Unlike GABA_A_ agonists that work directly on the receptor, BDZ increase the frequency of GABA_A_ channel opening, depending only on the endogenously available GABA [8,9,10]. Due to controlled neuronal inhibition and lower CNS depression risk, BZD rapidly replaced older medications such as barbiturates, meprobamate and chloral hydrate, becoming the most prescribed drug class in the world during the 1970s [11,12]. Although they possess a high therapeutic index, BZD also come with several side effects, such as drowsiness, dizziness, fatigue, dysarthria, loss of coordination, headache and amnesia, and they have the potential of being addictive [5]. Their use was recommended for a short treatment, i.e., 4–6 weeks for insomnia, but physicians prescribed BZD for months or years, with patients finding it difficult to stop taking these medications because of withdrawal symptoms [13,14,15,16]. Controlled clinical trials confirmed that long-term administration produced tolerance and dependence [17,18]. Due to this considerable risk of abuse, in February 1984, the United Nations Commission on Narcotic Drugs placed 33 commercially available BZD under Schedule IV of the 1971 Convention on Psychotropic Substances [19,20,21,22,23]. BZD are abused at supratherapeutic doses to reinforce opioid euphoric effects and to alleviate the “crash” following stimulant abuse, or they are administrated to perpetrate drug-facilitated sexual assault, exploiting their hypnotic and amnestic side effects [16,24,25,26]. High BZD doses in combination with opioids or other CNS depressants increase the risk of death by suppression of medullary respiratory centers [27,28,29]. According to the United Nations Office of Drugs and Crime (UNODC) and the European Monitoring Centre for Drugs and Drug Addiction (EMCDDA), the concomitant non-medical use of opioids and BZD was further exacerbated by the increasing emergence of designer BZD (DBZD) [30,31].

The term “DBZD” is a misnomer, as the class also includes BZD marketed in only some countries, metabolites of registered BZD and structural analogues of therapeutically approved BZD [32,33]. These new psychoactive substances (NPS) have the same chemical structure as legal BZD, with an aromatic ring fused to a 1,4-diazepine ring and an aryl group in position R5 [34,35,36,37]. Slight alterations of the BZD core at different positions generated a large number of designer compounds, mainly 1,4-benzodiazepines, triazolobenzodiazepines and thienotriazolodiazepines. [6,38,39]. The newest DBZD have a triazolo ring fused to the 1,4 diazepine core and electron-withdrawing groups (bromine, chlorine, nitro etc.) in position R8 that increase the affinity for the GABA_A_ receptor [40,41].

Compared with classical BZD, these compounds produce strong sedation and amnesia, and they increase the risk of respiratory depression and death when used in combination with other CNS depressants [41,42]. However, they are illicit, with a relatively short life cycle in the NPS market, the majority of DBZD have not undergone clinical trials and our knowledge of their pharmacokinetics and toxicity is lacking and limited to self-reported experiences [43,44]. These substances are illegally manufactured, sometimes mimic legal medicines’ appearance, and are purchased inexpensively on the underground drug market through online platforms that facilitate anonymous trading and bypass regulatory systems [45,46]. Phenazepam and nimetazepam were the first DBZD identified in Europe on the internet in 2007, followed by etizolam in 2011 [47]. They are not strictly considered DBZD since they are approved for medical use in certain countries, but they have been implicated in several drug-related deaths in the United Kingdom between 2012 and 2013 [39,47]. In 2012 in Finland, pyrazolam, the first true DBZD not approved in any jurisdiction, was identified [48]. About thirty different DBZD have been reported to date to the UNODC Early Warning Advisory (EWA), with the majority of notifications received from European Countries [30,49,50,51,52,53]. According to the UNODC, bulk materials from India and China are brought into Europe where they are further processed and sold as fake alprazolam or diazepam [54]. Counterfeit Xanax (alprazolam) and erimin-5 (nimetazepam) tablets containing etizolam, flualprazolam and phenazepam were also seized in the United States (US), Australia, Singapore and Malaysia [30,55,56].

The misuse of DBDZ in conjunction with other drug use is a growing and widespread world health and safety concern [47,57,58]. The number of DBZD seizures and undercover purchases increased in the US from 2391 in 2018 to 6194 in 2019 according to the US National Forensic Laboratory Information System [59,60,61,62]. In 2020 amid shortages of classic drugs of abuse following COVID-19 restrictions, some drug users shifted from prescription sedatives to DBZD and novel synthetic opioids (NSO) [63,64,65,66]. Produced in clandestine laboratories, DBZD do not meet the same strict approval requirements as legal pharmaceuticals and may contain variable amounts of active ingredients or contaminants, i.e., NSO and other NPS [54]. Users generally are unaware of the presence of contaminants in a product, resulting in an increasing number of adverse health events for DBZD, including emergency room admissions and death investigations [67,68,69]. There is also increasing DBDZ prevalence in driving impairment and road traffic crashes [70,71]. According to the UNODC, between 2019 and April 2020, DBZD were identified in 48% and 83% of post-mortem and Driving Under the Influence of Drug (DUID) cases, respectively, with flualprazolam, flubromazolam and etizolam as the most frequently detected substances [54,72].

Due to the high abuse potential and life-threating consequences of DBZD use, between 2020 and 2021 clonazolam, diclazepam, etizolam, flualprazolam and flubromazolam were listed in Schedule IV of the Convention of Psychotropic Substances of 1971 [73]. Based on this public health risk, this scoping review reports the most recent emergency department (ED) admissions, DUID and postmortem investigations involving DBZD, with the objective of providing useful and updated toxicology and epidemiology data about DBZD intake to improve public health and safety efforts.

## 2. Results

**Figure 1:**
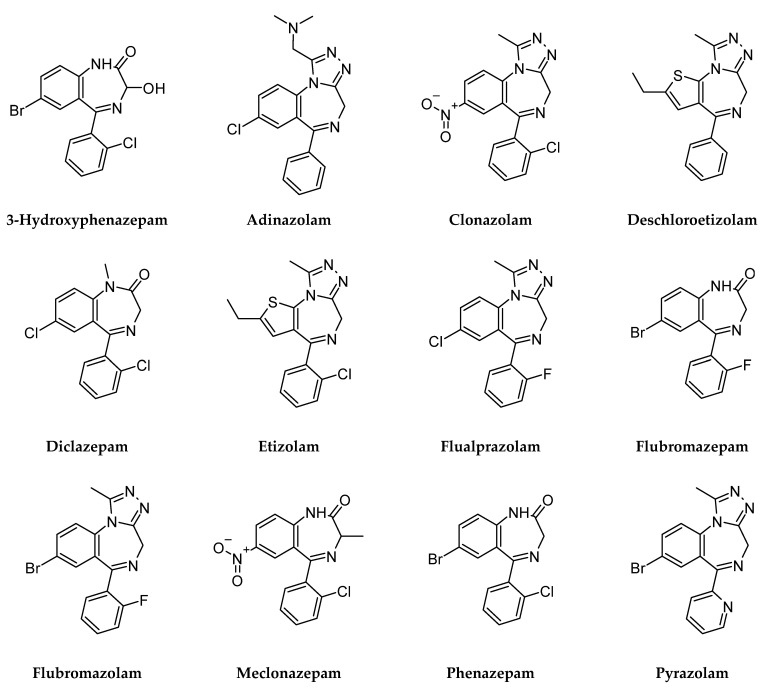
Chemical structure of “designer” benzodiazepines.

Of 372 potentially relevant reports, 324 were excluded because they did not describe ED admissions, DUID or fatalities associated with DBZD use. No relevant reports were found for 4-chlorodiazepam, alprazolam triazolobenzophenone derivate, bentazepam, bromazolam, cinazepam, clobromazolam, cloniprazepam, difludiazepam, fluclotizolam, flunitrazolam, fonazepam, methylclonazepam, metizolam, nifoxipam, nimetazepam, nitrazolam, norfludiazepam, tofisopam or thionordazepam, which were therefore excluded from the results. In 49 reports 3-hydroxyphenazepam, adinazolam, clonazolam, etizolam, deschloroetizolam, diclazepam, flualprazolam, flubromazepam, flubromazolam, meclonazepam, phenazepam and pyrazolam were the sole or explicit contributory cause of poisoning, driving-impairment and death. These DBZD were included in this study (Figure 1).

A total of 254 cases describing 1 drug offense, 2 self-administration studies, 3 outpatient department admissions, 44 ED admissions, 63 DUID and 141 deaths, reported between 2008 and 2021, are summarized in Table 1. Age, sex, observations (i.e., symptoms, death scene information etc.), detected concentrations in biological matrices and co-exposure concentrations are also displayed.

Most patients and victims were young individuals of both sexes, often with a previous history of substance abuse and mental illness. Acute intoxications and deaths related to DBZD, alone or in combination with other drugs of abuse, were reported in Finland, Germany, Japan, Norway, Poland, Sweden, UK and USA. DBZD were screened using LC-HRMS (LC-QTOF-MS and LC-Orbitrap-MS) and quantified with LC-MS, LC-MS/MS, LC-DAD, GC-MS or GC-MS/MS.

### 2.1. Adinazolam

Adinazolam or 1-(8-chloro-6-phenyl-4*H*-[1,2,4]triazolo[4,3-*a*][1,4]benzodiazepin-1-yl)-*N*,*N*-dimethylmethanamine is a short acting triazolo-BZD with anxiolytic, antidepressant, anticonvulsant and sedative properties [121,122]. Clinical studies revealed that drowsiness and dizziness are commonly observed after oral administration of adinazolam up to 70 mg, resulting in significant amnestic and psychomotor effects at higher doses [123,124,125]. Adinazolam was never FDA approved and never introduced onto the public market; however, it started to emerge as an illegal designer drug in 2015 [126,127]. The first reported adinazolam-related death concerned a young woman found dead in her apartment next to five resealable bags with unidentified powders/crystals. In the US, since April 2020, adinazolam was identified in at least three toxicology cases in association with etizolam, fentanyl and flualprazolam [128]. One male, one female and one unknown sex individual, all of whom were aged 20–40 years and each either from Michigan, Mississippi or Rhode Island, were the decedents. Adinazolam was identified in postmortem blood samples but was neither quantified nor listed as the cause of death.

### 2.2. Clonazolam

6-(2-Chlorophenyl)-1-methyl-8-nitro-4*H*-[1,2,4]triazolo[4,3-*a*][1,4]benzodiazepine, also known as clonitrazolam, is the triazolo-analogue of clonazepam [1,129]. Clonazolam is described as “insanely powerful”, producing strong sedation and amnesia at oral doses as low as 0.5 mg, resulting in easy accidental overdose [78]. It was found for the first time in seized yellow capsules by Swedish police on October 2014 and reported to the EMCDDA on January 2015 [51]. Two patients were admitted to ED after consuming clonazolam bought on the Internet. Clonazolam was not confirmed, and the dose was estimated based on the patient’s self-report. In the other four cases, clonazolam or clonazolam and etizolam (one case) were identified. The primary adverse effect was CNS depression.

### 2.3. Deschloroetizolam

Deschloroetizolam is a short-acting thienotriazolodiazepine that differs from etizolam by the absence of a chlorine on the benzene ring with consequent reduced potency [1]. On 1 September 2014, the UK Focal Point reported that the substance was confirmed after analysis of a blue seized tablet [50]. There are few data available on deschloroetizolam. In a self-administration study, one of the authors ingested one-half pink tablet of deschloroetizolam, about 6 mg, bought on the Internet [79]. After 15 min, the subject’s overall behavior changed rapidly; both physical and cognitive effects were described. Oral fluid was collected after 30 min. Deschloroetizolam and diclazepam’s metabolites, lorazepam and lormetazepam, were detected in a young male. The subject was found dead with injection materials and several small plastic bags labelled with different DBZD [81].

### 2.4. Diclazepam

Diclazepam, or 2-Chlorodiazepam, is the 2’-chloro derivative of diazepam and the positional isomer of 4-chlorodiazepam [84]. It was reported to EMCDDA by Germany in August 2013 [49]. In two of three cases displayed, subjects were admitted to the ED in a severe state of agitation and disorientation; diclazepam was detected along with stimulants and dissociatives. In the third ED admission, diclazepam was the sole drug reported. Symptoms of intoxication were mainly characterized by CNS depression and a withdrawal syndrome. The patient reported having ingested two 30 mL vials of 4 mg/mL diclazepam (240 mg) purchased online. Again, 13 drivers apprehended for DUID submitted to a clinical test of impairment (CTI). The level of impairment was assessed based on the single test results and the individual’s general condition. Common signs of impairment were found for alertness, appearance, cognitive function, motor coordination and vestibular function. Heide et al. report four additional DUID cases. Subjects were aged between 30 and 39 years; sex was not specified, and diclazepam was found in blood at concentration ranging from 5.4 ng/mL to 32 ng/mL [86]. The subjects did not show impairment. The only death reported involved a young man with a history of methamphetamine use found deceased at home. He previously told a friend that at times he took etizolam. Retrospective quantitative analysis revealed the presence of diclazepam and flubromazolam, along with opioids and stimulants. In addition, in 2013, a French patient was admitted to the ED after ingestion of two pills labelled “diclazepam” and “2-aminoindane” bought on the Internet. Upon clinical examination, the patient was anxious, but the anxiety resolved, and the patient was discharged the same day [130]. Diclazepam was neither confirmed nor quantified.

### 2.5. Etizolam

Etizolam, or 4-(2-chlorophenyl)-2-ethyl-9-methyl-*6H*-thieno[3,2-*f*][1,2,4]triazolo[4,3-α][1,4]diazepine, is a short-acting thienotriazoldiazepine introduced in 1983 under the trade name Depas^®^ [131,132]. It is currently used in India, Italy, Japan and Korea for the short-term treatment of insomnia, anxiety and panic attacks, but it is not approved for medical use elsewhere [55,71]. It was reported to EMCDDA in December 2011 by UK [133]. Three intoxications required ED admission. Three children were found drowsy and wobbly after eating colored pills thought to be candies. Etizolam was confirmed in one patient’s urine. In addition, a subject was found unconscious next to a syringe of heroin. He had previously ingested a large quantity of etizolam tablets. Three patients with psychiatric disorders presented at an outpatient department for etizolam detoxification after exhibiting tolerance and withdrawal. Etizolam was prescribed or illegally obtained in one case and was taken at supratherapeutic doses. For six DUID cases, three were apprehended drivers undergoing CTI, while three drivers were stopped for impaired driving and underwent a standardized field sobriety test (SFST). These results supported the diagnosis of motor and functional impairment. The other two males, ages 34 and 19 years, underwent CTI [85]. Etizolam was found in blood at concentrations of 31 ng/mL and 120 ng/mL, respectively; however, impairment was impossible to determinate or not reported. A total of 34 deaths were reported. In five cases, etizolam was found in association with diclazepam, (one case), flubromazepam (one case), flubromazolam (two cases) and flualprazolam and flubromazolam in one case. In 33 cases the cause of death was reported as accidental overdose due to polydrug toxicity; subjects were known drug users or had a history of mental disorders. In the remaining case [92], the subject was found dead in the bathroom with a suicide note in her diary. In these nine cases [92,95,97], etizolam was detected in peripheral blood at concentrations of 1–237 ng/mL. Subjects were seven males and two females between 22 and 61 years of age, residing in Japan, the UK or the US. However, etizolam was not listed as the cause of death.

### 2.6. Flualprazolam

Flualprazolam is the ortho fluorine analogue of alprazolam that was reported to the EMCDDA by Swedish police in January 2018 [99]. Seven young patients were transported to the ED after ingesting a BZD thought to be alprazolam. Three patients exhibited sedation and verbal impairment, two CNS depression, and two were asymptomatic. In three cases the presence of flualprazolam was not confirmed. Another thirteen DUID cases were reported. One individual was subjected to the CTI while twelve other drivers underwent SFSTs. Considerable motor and functional impairment were observed. Two biological samples screened positive for etizolam. Furthermore, Papsun et al. reported an additional 11 DUID [101]; however, demographic information and flualprazolam blood concentrations were not available. A total of 38 deaths were reported. All cases had multiple drugs; one was also positive for etizolam. In 36, the cause of death was listed as accidental overdose due to multiple drug toxicity, while in 2 cases they were ruled intentional flualprazolam poisonings. Furthermore, there were 28 additional deaths in which flualprazolam was not listed as the cause of death; these include 5 decedents from Finland, 13 from Sweden and 10 from the US. Flualprazolam blood concentrations ranged from 3 ng/mL to 620 ng/mL [101,102].

### 2.7. Flubromazepam

7-Bromo-5-(2-fluorophenyl)-1,3-dihydro-2*H*-1,4-benzodiazepin-2-one, well known as flubromazepam, was detected for the first time in ten seized capsules in Germany and reported to the EMCDDA in March 2013 [49]. Four subjects were admitted to the ED in a profound state of agitation and delirium, followed by rigidity and CNS depression. In one case, flubromazepam’s depressant effect was mitigated by the presence of methoxyphenidine. Only one DUID was reported. The driver was mildly impaired based on the CTI. Another apprehended, a 22-year-old driver, had a flubromazepam blood concentration of 7 ng/mL but did not show impairment on his CTI [86]. Only a single death case is included for flubromazepam. This young man was admitted to the ED in a severe state of CNS depression requiring resuscitation and mechanical ventilation; he died after six days of hospitalization. Flubromazolam and U-47700, which was also detected, were listed as the cause of death.

### 2.8. Flubromazolam

Flubromazolam is the triazolo-derivate of flubromazepam. It was identified in Sweden in 10 seized white tablets labelled “XANAX” and reported to EMCDDA in October 2014 [50]. It possesses strong and long-lasting depressive effect on the CNS. Eighteen patients were admitted to the ED in a severe state of CNS depression with functional and motor impairment. In 16 cases, flubromazolam was the sole drug detected, while in 2 cases subjects were also positive for meclonazepam. One patient required three days of hospitalization. After logical verbal contact was established, he admitted that he bought flubromazolam on the Internet and consumed about 3 mg approximately 19 h before ED admission [106]. Eleven flubromazolam DUID cases were reported; in two, driving impairment was assessed by CTI, while in the remaining nine, a SFST was performed by officers. Motor and functional impairment was evident in all subjects. Flubromazolam was listed as a contributory cause of death in four cases. Abdul et al. reported two additional deaths in which flubromazolam was found in femoral blood at concentrations of 8 and 16 ng/mL [108]. The two male decedents were 32 years old and 46 years old. The cause of death was not flubromazolam toxicity. Flubromazolam pharmacokinetics were assessed in a self-administration study. One of the authors ingested a 0.5 mg capsule of flubromazolam. During the following 24 h, the author observed strong sedation and considerable memory impairment.

### 2.9. Meclonazepam

Meclonazepam is structurally related to clonazepam and was reported for the first time to EMCDDA in August 2014 after identification in 145 seized capsules in Sweden [50]. A young man was admitted to the ED in December 2014 after ingesting approximately 100 tablets (600 mg) of meclonazepam. The subject was awake but not completely lucid.

### 2.10. Phenazepam and 3-Hydroxyphenazepam

Phenazepam, also known as “Bonsai”, “Zannie” or “Supersleep”, is a long-acting benzodiazepine developed in the 1970s and currently used as an anxiolytic, hypnotic and for the treatment of Alcohol Withdrawal Syndrome in the former USSR [134]. Phenazepam was reported to EMCDDA in July 2011 by Germany and UK. It is metabolized to the active metabolite 3-hydroxyphenazepam by different isoforms of CYP450 [114,135]. 3-Hydroxyphenazepam was identified in a seized white tablet and reported in October 2016 by Denmark. Three subjects were admitted to the ED after ingesting illicit phenazepam purchased on the Internet. Patients exhibited both motor and functional impairment and depressant effects. One patient had Asperger’s syndrome [110]. In May 2016, a patient was admitted to the ED after ingesting four tablets of 3-hydroxyphenazepam. There also are 19 DUID and a drug offense cases included in Table 1. Of these, 11 underwent SFST, 5 had roadside drug tests, 3 CTI, while 1 driver refused to perform SFST, and symptoms of impairment were provided by the officer’s observations. Moderate to considerable motor and functional impairments were evident in all drivers. Heide et al. reported one additional DUID of a young driver submitted for CTI [86] who also had a phenazepam blood concentration of 120 ng/mL. The driver passed his CTI and was declared not impaired. Of sixty deaths reported, phenazepam alone was listed as the sole cause of death in two cases, while the remaining were attributed to accidental overdose due to polydrug toxicity.

### 2.11. Pyrazolam

Pyrazolam is the triazolo analogue of bromazepam that was identified in Finland in 10 white tablets and notified to EMCDDA in August 2012 [136]. In February 2016, a young man was found dead in an advanced state of putrefaction next to five plastic bags labelled pyrazolam, diclazepam, 3F-phenmetrazine, 1-(2-fluorophenyl) propan-2-amine and diphenhydramine hydrochloride, as well as one unlabelled bag. Asphyxia promoted by polydrug intoxication was listed as the cause of death.

## 3. Discussion

Seventy percent of the new DBZD were introduced into the European Union (EU), representing about thirteen percent of worldwide NPS seizures [137]. The EU market is dominated by a handful of these, most notably clonazolam, diclazepam, etizolam, flualprazolam, flubromazolam and phenazepam [31,58,64,138,139,140]. Etizolam, in particular, is the “street” BZD that is most often implicated in drug related deaths. In Scotland, its numbers grew from 223 in 2016 to 752 in 2019 [141]. DBZD are a worldwide growing public health concern. In the US, more than 5000 cases regarding clonazolam, etizolam and flualprazolam were reported in the US NFLIS from Federal, State and local laboratories between October and December 2020 [142]. The Center for Forensic Sciences Research and Education confirmed this trend for the first quarter of 2021, underlining the popularity of flubromazolam [143]. Etizolam, flualprazolam and flubromazolam were recently identified in counterfeit Xanax tablets in Canada, and their use is increasing also in Central and South America, mainly in Brazil, Chile and Paraguay [54,144]. Surprisingly, no updated data on DBZD are available from Asia, although most NPS are synthesized in this area of the world. However, a small number of DBZD may be sourced from companies in India, typically as finished medicinal products [54,145,146,147].

According to the UNODC, the highest public health risk around the world is from etizolam, flualprazolam, flubromazolam and phenazepam [54,72]. DBZD are widely available on the Internet in different forms, i.e., blotters, liquids, pills, powders and tablets, and sold at low prices [148]. Etizolam and phenazepam are further diverted from the regulated market and illegally imported from those countries where they are licensed therapeutic drugs [138,149]. For most NPS placed under international control, the number of reports decreased rapidly the year after the scheduling decision [150]. However, for flualprazolam, phenazepam, flubromazolam and etizolam, enforcement was delayed two, five, seven and nine years, respectively, after formal notification [73]. The social harms produced by these drugs’ long residence on the illicit market are characterized by an increasing rate of DBZD-related deaths, involvement of criminal activity, violence, risk-taking behavior, suicide attempts and concurrent substance use disorders [151,152].

Only cases in which DBZD were the sole or a contributory cause of intoxication, impairment or death are included in Table 1, which evaluates global DBZD intake. This facilitates review of the biological concentrations in the different types of cases. Clinicians are unaware of DBZD and their contribution to drug overdoses and deaths, sometimes leading to incorrect interpretations of cause of death. Clinicians should be asking patients about substance abuse including NPS and DBZD during routine preventive care and ED visits. The patients may not be aware of the identity or concentration of DBZD in a drug product before suffering symptoms of intoxication [135]. When a DBZD is the only drug identified, it provides the opportunity to characterize its associated sedative-hypnotic toxidrome as seen in cases [45,74,77,79,80,82,84,85,86,89,90,91,98,106,109,111,113,115].

However, since few pharmacokinetics studies were performed [82,109], it is currently hard to associate concentrations in biological matrices with presumable related adverse-effects. To date, correlations between dose and response, duration of action, metabolism, and onset of action are still poorly understood, making it harder for users to accurately dose the compound they purchased, increasing the prospect of potential intoxication. The slow elimination and the hepatic transformation in active metabolites of certain DBZD (i.e., flubromazolam and phenazepam) are responsible of their accumulation in lipid-based tissues, which can lead to a delayed overdose in cases of repeated consumption [44,82,91,152,153]. There was overlap between diclazepam, etizolam and phenazepam blood concentrations in impaired and non-impaired drivers [85,86]. Similarly, blood etizolam and flualprazolam concentrations were similar in DUID cases and deaths [86,92,101,102]. This may reflect differences in tolerance that appear after frequent drug exposure. In other cases, there is too little information or analytical data to improve our knowledge about the DBZD [74,83,104], and in many cases, because polypharmacy is the rule rather than the exception, it is not possible to assign causation to a single drug because the death is due to the drug combination [78,86,88,100,101,112]. On the other hand, it is also possible that many individuals exposed to DBZD never developed significant adverse events [154]. However, a major problem is knowing that in many cases the DBZD will never be detected due to a lack of analytical method capability or even just to unawareness of the presence of this class of NPS. Furthermore, the newest DBDZ may have high cross-reactivity with common BZD immunoassays, which often do not distinguish between designer and prescribed BZD. Metabolism to licensed BZD, the sale of metabolites of prescribed BZD and the unavailability of confirmatory testing in health care centers pose the risk of an incorrect interpretation of analytical findings [5,127,155,156,157]. The roles DBZD play in deaths remains poorly understood, and how different pathologists and toxicologists attribute and interpret cause of death is largely unknown. For attributing the cause of death, each case must be assessed individually, taking into account the circumstances surrounding the death, drug tolerance and postmortem redistribution. [119,158,159]. The present data should inform interpretation of DBZD-related deaths and apprise law enforcement, clinicians and ED personnel on the dangers of DBZD.

## 4. Materials and Methods

31 DBZD were selected after consulting the UNODC Early Warning Advisory on NPS portal, the European Database on New Drugs, the US National Poison Data System and the Japanese Data Search System for NPS. Thereafter, a comprehensive literature search was performed using PubMed, Scopus, Google Scholar and Web of Science bibliographic databases to identify scientific reports on ED admissions, DUID and fatalities associated with DBZD use. Database-specific search features with truncations (represented by an asterisk) and multiple keywords (represented by quotation marks) were employed. The search terms employed were: acute, abuse, “access* to emergency department”, “adverse effect*”, diversion, “driving under the influence of drug*”, DUID, fatal, “illegal market”, intoxication*, lethal, misuse, overdose*, prescription, poison*, report*, schedule*, seizure* or traffic in combination with 3-hydroxyphenazepam, 4-chlorodiazepam, adinazolam, alprazolam triazolobenzophenone derivative, bentazepam, bromazolam, cinazepam, clobromazolam, cloniprazepam, clonazolam, deschloroetizolam, diclazepam, etizolam, flualprazolam, flubromazepam, flubromazolam, fluclotizolam, flunitrazolam, fonazepam, meclonazepam, metizolam, methylclonazepam, nimetazepam, nifoxipam, nitrazolam, norfludiazepam, norflunitrazepam, phenazepam, pyrazolam, thionordazepam or tofisopam. Further studies were retrieved from the reference list of selected articles and from reports from international institutions such as the World Health Organization (WHO), the EMCDDA, the US Drug Enforcement Administration (DEA) and the US Food and Drug Administration (FDA). Articles written in English and only one in Swedish were included. Databases were screened through March 2021 and references were independently reviewed by one of the authors to determine their relevance to the present article.

## 5. Conclusions

The outbreak of DBZD is a rising health and social concern. Clinical and forensic toxicologists are on the front line, in cooperation with public health safety institutions, to identify emerging DBZD in cases of intoxication, drug offenses and unexplained deaths. In order to decrease the availability of these substances in the global illicit drug market, more effort is needed by early warning agencies to reduce the timing between formal notifications and scheduling decisions. Further studies, professional training and analytical development are required to reduce the undercounting and underreporting of the cases in order to obtain robust and consistent epidemiological data.

## Figures and Tables

**Table 1:** Designer benzodiazepine (DBZD) case reports.

Compound	Study	Age; Sex	Observations	Concentration ^†^/Dose	Co-Exposure Concentration(s) ^†^	Ref.
3-Hydroxyphenazepam	ED	29; M	Tremor	Urine screen +	-	[74]
Adinazolam	Death	24; F	Multiple drugs	Blood 18 Urine 82.1	U-47700 blood 1470, urine 3940 SRT blood 89.5, urine 32.5 N-Ethylhexedrone blood 58.1, urine 14 4-CIC blood 8, urine 130 4-CMC blood 1.7, urine 417	[75]
Clonazolam	ED	25; M	Agitation, Aggressivity	100 mg	BZD urine screen + THC urine screen +	[76]
		28; M	Lethargy	≅15 mL of a 0.4 mg/mL solution	-	[77]
		26; M	Respiratory depression, Unconscious	Serum 6	MDZ urine screen + U-47700 serum 351 THC serum 3.3, urine screen + THCCOOH serum 121.6, urine screen + CIT urine screen +	[78]
		34; M	Confusion, Lethargy	Serum 10.2	Etizolam serum 281	[79]
		20; M	Ataxia	Urine screen +	MXE urine screen +	[74]
		26; F	Coma	Blood 77 (4 h) Blood 15 (8 h) Blood 9 (12 h)	-	[80]
Deschloroetizolam	Death	31; M	Multi-organ congestion	Blood 11 Urine screen +	LMZ urine 258 LZP urine 115 OXZ urine 17.4 THC urine screen +	[81]
	Self-administration	56; M	Dizziness, Fatigue, Language disorder, Difficulty concentrating; Took 6 mg	Oral Fluid 6.5 (30 min)	-	[82]
Diclazepam	ED	30; M	Agitation, Confusion, Disorientation, Inability to communicate, Muscular rigidity, Myosis, Tachycardia, Tachypnoea	Plasma 3.5	DIP plasma 308, urine 631 MPH plasma 3 THCCOOH urine 120	[83]
		39; M	Agitation, Dilated pupils, Tachycardia	Urine screen +	3,4-CTMP	[74]
		30; M	Mydriasis, Respiratory depression, Unconscious, Withdrawal syndrome.	240 mg	-	[84]
	DUID	18; M	Considerable impairment	Blood 57	-	[85]
		27 *; Not reported	Moderate impairment	Blood 61	EtOH blood 0.053 g/L	[86]
		32 *; Not reported		Blood 45	EtOH blood 0.084 g/L	
		22 *; Not reported		Blood 32	-	
		<20; Not reported		Blood 19	-	
		47 *; Not reported		Blood 16	LZP blood 63	
		52 *; Not reported		Blood 11	NZP blood 17	
		22 *; Not reported	Mild impairment	Blood 35	LZP blood 14	
		22 *; Not reported		Blood 7.7	THC blood 0.7	
		22 *; Not reported		Blood 5.1	-	
		37 *; Not reported	Considerable impairment	Blood 48	-	
		27 *; Not reported		Blood 35	THC blood 1.1	
		32 *; Not reported		Blood 14	-	
	Death	28; M	Multiple drugs	Blood 70	Flubromazepam blood 10 U-47700 blood 330 MAMP blood 290 AMP blood 150 DOC blood screen +	[87]
Etizolam	ED	31; M	Bradypnea, Unresponsive	Serum 103	6-AM urine 272 MOR urine 1000 COD urine 322	[88]
		6; M	Ataxia, Drowsiness, Mydriasis	-	-	[89]
		9; M		-	-	
		10; M		Urine screen +	-	
	OD	23; M	Tolerance, Withdrawal syndrome	2.5 mg/day for 1 month	-	[90]
		32; M	Catatonia, Withdrawal syndrome	4 mg/day for 2 months, abruptly stopped	-	[91]
		30; M	Bradypnea, Loss of consciousness, Seizures, Withdrawal syndrome	Took 50 mg/day to 100 mg/day for several months Urine screen +	LZP urine screen +	[45]
	DUID	27 *; Not reported	Mild impairment	Blood 210	-	[86]
		<20; Not reported		Blood 120	TMD blood 71	
		42 *; Not reported	Considerable impairment	Blood 110	-	
		37; M	Delayed comprehension and reaction time, Impairment, Incoordination, Lethargy	Blood 40	AMP blood screen +	[71]
		20; F		Blood 88	THC blood 11	
		35; M		Blood 330	MAMP blood screen + AMP blood screen +	
	Death	59; F	Suicide	Blood 264	αOH-Etizolam blood 9.4 8OH-Etizolam blood 9.3	[92]
		42; M	Multiple drugs	Blood 86	PB blood 5082, urine 1736 PMZ blood 107, urine 806 CPZ blood 144, urine 1437	[93]
		42; M	Multiple drugs	Blood 300 Urine 100	MDVP blood 46, urine 1300 PEN blood 160, urine 1200 EPH blood 68 OLZ blood 4200 MIR blood 570	[94]
		48; M	Accidental death, Multiple drugs	Serum 4	MTD serum 381 EDDP serum 86 MOR serum 290 COD serum 47 PGB serum 14 PAR serum screen +	[95]
		40; M		Serum 17	MOR serum 44 COD serum 7 COC serum screen + BE serum 1536	
		29; M		Serum 40	DZP serum screen + Nor-DZP serum 18 OXZ serum screen + MTD serum 133 EDDP serum 7 THC serum 2.4 THCCOOH serum 17 PGB serum 19 CYC serum 78	
		38; M		Serum 44	DZP serum 55 Nor-DZP serum 131 OXZ serum 11 MTD serum 886 EDDP serum 121 SRT serum 6 PMZ serum 57 PGB serum 13000	
		48; M		Blood 4	DZP blood 99 Nor-DZP blood 316 TMZ blood 15 OXZ blood 29 MOR blood 6 COD blood 83 AMP blood 394 AMI blood 307 NTP blood 283 PAR blood screen +	
		34; M		Blood 8	Diclazepam blood 2 COD blood 108 CIT blood 423 Nor-CIT blood 93	
		23; M		Blood 8	EtOH blood 0.77 g/L ALP blood 300 Nor-DZP blood 5 MOR blood 5 COD blood 16 BE blood screen + SRT blood 19 PPL blood 8	
		55; M		Blood 7	DHC blood 1681 COC blood 317 BE blood 5135 AMI blood 1859 NTP blood 582 PGB blood 22300	
		39; M		Blood 45	MTD blood 377 COC blood 18 AMI blood 885 PGB blood 6500000	
		38; M		Blood 172	DZP blood 6 Nor-DZP blood 22 LZP blood screen + MTD blood 1233 EDDP blood 129 MOR blood 16 COD blood screen + COC blood 10 BE blood 299 THCCOOH blood 11.2 MIR blood 27 PGB blood 35900	
		32; F		Blood 9	DZP blood 306 MTD blood 86 MOR blood 1292 COC blood 7 MIR blood 6 PAR blood 22000	
		43; M		Blood 93	DZP blood screen + ZPC blood 65 MTD blood 2297 COC blood screen + MIR blood 8 PGB blood 3700	
		42; M		Blood 85	DZP blood 16 MOR blood 880	
		37; M		Blood 85	MTD blood 189 PGB blood 8500	
		32; M		Blood 4	DZP blood 107 MOR blood 273 AMP blood 859 CPA blood screen + GBP blood 2600 PGB blood 10300 PAR blood screen +	
		35; M		Blood 16	MOR blood 269 COC blood screen + SRT blood 24 CBZ blood 2300 PGB blood 23,500	
		39; F		Blood 1	DZP blood 431 MTD v blood 634 PMZ blood 56 MIR blood 61 QTP blood 26 VPA blood screen + PGB blood 22,800 PAR blood screen +	
		32; M		Blood 18	DZP blood 131 MOR blood 34 DHC blood 6413 HCOD blood 96 AMI blood 310 PGB blood 10,200	
		49; M		Blood 1.5	Flubromazepam blood 33 DZP blood 89 MTD blood 685 MOR blood 44 MIR blood 12 PGB blood 38,100	
		54; F		Blood 12	DZP blood 90 MTD blood 973 TMD blood screen + COC blood 12 AMI blood 67 MIR blood 280 PGB blood 12,900	
		39; M		Blood 4	EtOH blood 0.24 g/L DZP blood 68 MOR blood 1076 COC blood 184 CE blood 22 MIR blood 121 QTP blood 16 PAR blood screen +	
		49; M		Blood 12	CZP blood screen + TAP blood 500 MOR blood 331 PGB blood 15,200 MIR blood screen +	
		28; M		Blood 29	EtOH blood 1.1 g/L DZP blood 16 THC blood 57.5 MIR blood 39 PGB blood 2900	
		39; M		Blood 3	DZP blood 238 LZP blood 10 MOR blood 75 SRT blood 92 GBP blood 6700 PRO blood 598 PAR blood 15,700	
		33; M		Blood 14	Flubromazolam blood 1 DZP blood screen + MOR blood 56 COC v blood 46 PRO blood 186 CLO blood 2060 Nor-CLO blood 1629 MIR blood 257 LTG blood 5800 GBP blood 24,600 PAR blood screen +	
		49; M	Accidental death, Multiple drugs	Blood 770 Central blood 2820 Hair 0.107	EtOH blood 0.19 g/L THCCOOH urine 192 THC hair 0.19 ng/mg AMP hair 3.37 ng/mg CAF blood 190,000 COC hair 0.22 ng/mg BE hair 0.068 ng/mg	[96]
		29; M	Accidental death, Multiple drugs	Central blood 45 Urine 13 Vitreous humor screen +	EtOH c blood 0.023 g/L, vitreous humor 0.014 g/L ALP c blood 228, urine 238, vitreous humor 17 α-OH-ALP c blood and urine screen + Nor-DZP c blood, urine and vitreous humor screen + FEN c blood 6, urine and vitreous humor screen + Nor-FEN c blood and urine screen + CDP c blood screen + DOX urine and vitreous humor screen +	[97]
		34; M		Blood 9 Central blood screen + Urine screen + Vitreous humor screen +	EtOH blood 0.023 g/L, vitreous humor 0.028 g/L Nor-DZP blood and urine screen + Desalkyl-FZP blood, urine and vitreous humor screen + 6-AM blood 11, c blood, urine and vitreous humor screen + MOR blood 185, c blood, urine and vitreous humor screen + COD p and c blood and vitreous humor screen + HCOD c blood and vitreous humor screen + CIT p and c blood, urine and vitreous humor screen + DPH p and c blood, urine and vitreous humor screen +	
		36; M		Blood 10 Urine 8 Vitreous humor screen +	Flubromazolam urine and vitreous humor screen + ALP blood 27, urine and vitreous humor screen + α-OH-ALP urine and vitreous humor screen + 7-Amino-CZP urine screen + FEN blood 31, vitreous humor screen + Nor-FEN blood and vitreous humor screen + MTD blood and vitreous humor screen + EDDP blood and vitreous humor screen + MAMP blood 1212, vitreous humor screen + AMP blood and vitreous humor screen +	
		28; M		Blood 15 Central blood 15 Urine 20	ALP blood 179, c blood 235, urine screen +, vitreous humor 92 DZP p and c blood, urine and vitreous humor screen + Nor-DZP p and c blood, urine and vitreous humor screen + TMZ c blood and urine screen + Nor-FEN c blood and vitreous humor screen + HCOD urine screen + MAMP p and c blood, urine and vitreous humor screen + AMP c blood, urine and vitreous humor screen + BE urine screen + DOX p and c blood, urine and vitreous humor screen + PMZ c blood and urine screen + CPA p and c blood, urine and vitreous humor screen +	
		30; M		Blood 187 Central blood 214 Urine 64 Vitreous humor 33	EtOH blood 0.002 g/L, vitreous humor 0.003 g/L Flualprazolam p and c blood, urine and vitreous humor screen + Flubromazolam blood 619, c blood 878, urine 552, vitreous humor screen + ALP p and c blood, urine and vitreous humor screen + DLP p and c blood, urine and vitreous humor screen + LZP p and c blood and urine screen + 7-Amino-CZP urine screen + FEN blood 17 Nor-FEN p and c blood, urine and vitreous humor screen + MAMP p and c blood, urine and vitreous humor screen + AMP p and c blood, urine and vitreous humor screen +	
Flualprazolam	ED	16; M	Lethargy, Slurred speech	Urine 72.1	Nor-DZP urine screen + THC-COOH urine screen +	[98]
		16; F		Urine 3	Nor-DZP urine screen +	
		16; M	CNS depression, Mild respiratory depression	Blood 14.6 Urine 19.4	Nor-DZP urine screen +	
		18; M	Unconscious	Blood 8	COC blood screen + THC blood screen +	[99]
	DUID	37 *; Not reported	Considerable impairment	Blood 15	TMD blood 65	[86]
		Not reported	Considerable impairment	Blood 4.3	DZP blood 25 BRP blood 1 FEN blood 6.2 COC blood 57	[100]
		31; M	Delayed comprehension and reaction time, Driving impairment, Incoordination, Lethargy	Blood 4.4	THC-COOH blood screen + LEV blood screen +	[101]
		22; M		Blood 8.3	EtOH blood 0.01 g/L	
		31; M		Blood 8.9	Etizolam blood screen + ALP blood screen + DLP blood screen + Nor-BUP blood screen + THC blood screen +	
		51; M		Blood 10	OXY blood screen + OXM blood screen +	
		47; M		Blood 11	CFN blood screen + FEN blood screen + MTD blood screen + COC blood screen +	
		24; M		Blood 13	-	
		30; M		Blood 39	BE blood screen + MTG blood screen +	
		20; M		Blood 46	EtOH blood 0.003 g/L	
		40; M		Blood 46	BPP blood screen +	
		20; M		Blood 65	THC blood screen +	
		26; M		Blood 68	Etizolam blood screen + MTD blood screen +	
	Death	28 *; Not reported	Suicide	Blood 28 ng/g	MIR blood 200 ng/g VEN blood 520 ng/g	[102]
				Blood 68 ng/g	EtOH blood 0.04 g/L BUP blood 0.94 ng/g, urine 17 ng/g Nor-BUP blood 0.83 ng/g, urine 15 ng/g	
			Suspected overdose, Multiple drugs	Blood 4 ng/g	VEN blood 1300 ng/g PGB blood 16000 ng/g	
				Blood 18 ng/g	MTD blood 150 ng/g	
				Blood 17 ng/g	EtOH blood 0.67 g/L, urine 1.33 g/L BUP blood 2.8 ng/g, urine 90	
				Blood 19 ng/g	-	
				Blood 14 ng/g	LPM blood 60 ng/g	
				Blood 21 ng/g	-	
				Blood 11 ng/g	BUP blood 0.9 ng/g, urine 40 NBUP blood 0.2 ng/g	
				Blood 36 ng/g	BUP urine 120 ng/g NBUP urine 7.4 ng/g PGB blood 1700 ng/g	
				Blood 30 ng/g	EtOH blood 0.68 g/L BUP blood 1.1 ng/g, urine200 ng/g 3F-AMP blood 10 ng/g MAMP blood 190 ng/g AMP blood 1000 ng/g	
				Blood 13 ng/g	EtOH blood 1.9 g/L	
				Blood 33 ng/g	N-ethyl-3F-AMP blood screen + 3F-AMP blood screen +	
		53; M	Suspected overdose, Multiple drugs	Blood 50	FEN blood 3.4 Nor-FEN blood 0.36 4-ANPP blood screen + ITZ blood screen + BRP blood 10, urine 23 6-AM blood 1.5 MOR blood 66 COD blood 6.6 CIT/ESC blood 76	[100]
		45; M		Blood 2.5	FEN blood 5 4-ANPP blood screen + TMD blood 33 BRP blood 1, urine 1.9 THC blood 0.62	
		48; M		Blood 5.4	CZP blood screen + FEN blood 4.7 Nor-FEN blood 1.6 Acetyl-FEN blood 1.2 4-ANPP blood screen + BRP blood 0.1, urine 0.2 MOR blood 8 DPH blood 190	
		47; F		Blood 13	FEN blood 190 Nor-FEN blood 5.4 Acetyl-FEN 0.15 4-ANPP blood screen + BRP blood 6.7, urine 2.1 6-AM blood 12 MOR blood 85 COD blood 7 MAMP blood 580 AMP blood 55 XYL blood 170	
		53; M		Blood 20	FEN blood 19 Nor-FEN blood 4.2 4-ANPP blood screen + BRP blood 0.2 MOR blood 15 XYL blood 30	
		29; M		Blood 3.6	7-Amino-CZP blood 5.2 FEN blood 37 Nor-FEN blood 1.3 4-ANPP blood screen + TMD blood 70 BRP blood 1.1, urine 0.8 MAMP blood 42 AMP blood 10 DPH blood 490	
		22; M	Suspected overdose, Multiple drugs	Blood 3.2	EtOH blood 0.017 Desmethyl-LPM blood screen +	[101]
		53; M		Blood 2.1	FEN blood screen + MTD blood screen + COC blood screen + GBP blood screen +	
		32; M		Blood 2.2	BE blood screen + THC blood screen + MTG blood screen + CBP blood screen + HYZ blood screen + GBP blood screen +	
		29; M		Blood 4.1	ITZ blood screen + MAMP blood screen + AMP blood screen +	
		35; F		Blood 5.2	EtOH blood 0.008 g/L BE blood screen + THC blood screen +	
		38; M		Blood 6.2	ITZ blood screen + FEN blood screen + MAMP blood screen + AMP blood screen + HYZ blood screen +	
		23; F		Blood 9.9	FEN blood screen + 4-ANPP blood screen + BE blood screen + THC blood screen + MAMP blood screen + AMP blood screen +	
		23; M		Blood 15	FEN blood screen + 4-ANPP blood screen +	
		21; M		Blood 29	FEN blood screen + MAMP blood screen + AMP blood screen + THC blood screen +	
		36; M		Blood 63	MTD blood screen +	
		40; M	Suicide	Blood 26.5	DZP blood 9 Nor-DZP blood 4 MTD blood 736 EDDP blood 149 PGB blood 1900	[103]
		30; M	Suspected overdose, Multiple drugs	Blood 3	DZP blood screen + 6-AM blood screen + MOR blood 196 COD blood 11 THC blood screen + MIR blood screen + PGB blood 12000	
		44; M		Blood 35	DZP blood screen + MTD blood 549 MOR blood screen + COC blood screen + BE blood screen + MDMA blood 29 MDA blood screen + MIR blood 58 GBP blood screen + PGB blood 18,100	
		40; F		Blood 14.5	MTD blood 711 EDDP blood 67 4F-MDMB-BINACA blood screen + MDMB-4en-PINACA blood screen + MIR blood 3229 PGB blood 7900	
		37; M		Blood 14.1	Etizolam blood 85 CBZ metabolites blood screen + MTD blood 189 5F-AMB metabolites blood screen + THC metabolites blood screen + PGB blood 8500	
		51; M		Blood 3.1	ALP blood 68 DZP blood 367 Nor-DZP blood 364 OXZ blood 45 TMZ blood 19 MTD blood 694 EDDP blood 365 MOR blood 62 COD blood 14 BE blood screen + SRT blood 31 PGB blood 47,000 RSP blood 35	
		57; M		Blood 5.7	COC blood 41 BE blood 718 CIT blood 707	
		42; F		Blood 15.1	MOR blood 410 COD blood 19 PGB blood 9900	
		42; M		Blood 9	ALP blood 35 CZP blood 7 DZP blood 61 Nor-DZP blood 82 NZP blood 16 BUP blood 0.5 MOR blood 197 COD blood 11 COC blood screen + BE blood 258 MIR blood 23 PGB blood 900	
Flubromazepam	ED	25; M	Agitation, Aphasia, Ataxia, Confusion, Dysarthria, Hypertension, Hyposthenia	Blood 411	BZD urine screen + THC urine screen + MXP blood 247	[104]
		24; M	Agitation, Coma, Delirium, Mydriasis, Rigidity, Tachycardia, Tremor	Urine screen +	-	[74]
		47; M		Urine screen +	3OH-Flubromazepam urine screen +	
		45; M		Urine screen +	3OH-Flubromazepam urine screen +	
	DUID	37; M	Mild impairment	Blood 600	-	[85]
	Death	24; M	Apnea, Coma, Rattling breath, Hypothermia, Myosis, Tachycardia, Unconscious	Plasma 830	U-4770 plasma 370	[105]
Flubromazolam	ED	27; M	Coma, Cyanosis, Hypotension, Unconscious, Respiratory depression, Tachycardia	Serum 59 Urine 105	-	[106]
		20; M	Ataxia, Coma, Disorientation, Lethargy, Hallucinations, Hypotension, Miosis, Mydriasis, Seizures, Slurred speech, Tremor, Unconscious	Urine screen +	-	[74]
		18; F		Urine screen +	-	
		65; M		Urine screen +	-	
		26; M		Urine screen +	Meclonazepam urine screen +	
		15; F		Urine screen +	-	
		23; M		Urine screen +	-	
		49; M		Urine screen +	-	
		27; M		Urine screen +	-	
		20; F		Urine screen +	-	
		17; F		Urine screen +	-	
		17; F		Urine screen +	-	
		19; F		Urine screen +	-	
		23; M		Urine screen +	-	
		18; M		Urine screen +	Meclonazepam urine screen +	
		35; M		Urine screen +	-	
		18; M		Urine screen +	-	
		18; M		Urine screen +	-	
	DUID	20, M	Mild impairment	Blood 0.48	-	[85]
		19; M	Considerable impairment	Blood 100	-	
		17; M	Driving impairment, Lethargy, Lack of balance, Slurred speech	Blood 17	THC blood 6.1	[71]
		18; M		Blood 18	THC blood 2.2	
		21; M		Blood 19	BE blood 348 THC blood 1.5	
		17; F		Blood 14	EtOH blood 0.014 g/L	
		19; F		Blood 21	COC blood screen + BE blood 749	
		19; M		Blood 7	CZP blood 7 7-Amino-CZP blood 26 OXY blood screen + THC blood 27	
		22; F		Blood 12	THC blood 2.9	
		35; F		Blood 31	THC blood 4.1	
		21; F		Blood 8.2	BE blood 356 THC blood 1	
	Death	34; M	Multiple drugs	Blood screen +	DZP blood 200 Nor-DZP blood 180 TMZ blood 11 MAMP blood screen + AMP blood 70 3-FPM blood 2.4, central blood 2.6 AMI blood 440 NTP blood 290	[107]
		39; M	Multiple drugs	Blood 70	EtOH blood 0.24 g/L Etizolam blood 4 DZP blood 68 Nor-DZP blood 365 TMZ blood 6 OXZ blood 22 6-AM blood screen + MOR blood 1149 COD blood 289 COC blood 184 BE blood 525 CE blood 22 QTP blood 16 MIR blood 121	[108]
		49; M		Blood 33	Etizolam blood 1.5 DZP blood 89 Nor-DZP blood 575 OXZ blood 13 TMZ blood 5 MTD blood 685 EDDP blood 100 6-AM blood screen + MOR blood 73 COD blood 18 MIR blood 12 PGB blood 38.1	
		33; F		Blood 1	Etizolam blood 14 7-Amino-CZP blood screen + MOR blood 91 COC blood 46 BE blood 2573 CLO blood 2060 Nor-CLO blood 1629 GBP blood 24.6 LTG blood 5.8 PRO blood 186	
	Self-administration	44; M	Considerable impairment, delayed comprehension and reaction time, lethargy, muscle relaxation, partial amnesia, sedation	0.5 mg oral ingestion Serum 7.4 (5 h) Serum 8.6 (8 h) Serum 5.2 (30 h) Hair 0.44 pg/mg (2 w) Hair 0.60 pg/mg (4 w)	BZD urine screen +	[109]
Meclonazepam	ED	31; M	Agitation, Non-reactive pupils	Urine screen +	-	[74]
Phenazepam	ED	26; M	Ataxia, Lack of balance, Memory impairment, Slurred speech	Blood 1200	BZD urine screen + VEN blood screen +	[110]
		42; M	Confusion, Disorientation, Mydriasis	Blood 490	-	[111]
		29; M	Unresponsiveness, Tachycardia	Serum 1400	BZD serum screen + U-47700 serum 240	[112]
	Drug offense	22 *; Not reported	Moderate motor impairment	Blood 260	THC blood 0.7	[86]
	DUID	50; F	Behavioral aberrations, Moderate/considerable functional disorders	Blood 270	-	[113]
		27; M		Blood 310	-	
		21; M		Blood 3000	-	
		47; F		Blood 230	-	
		47; M		Blood 380	-	
		18; M	Agitation, Amnesia, Disorientation, Lack of balance, Lethargy, Mydriasis, Myosis, Non-reactive pupils, Sedation, Slurred speech, Slow reactivity, Tachycardia	Blood 180	THCCOOH blood 28	[114]
		27; M		Blood 500	CBP blood 6.1	
		22; M		Blood 750	TZD blood screen +	
		29; F		Blood 310	AMP blood 190 QTP blood screen +	
		39; M		Blood 170	THCCOOH urine screen +	
		23; M		Blood 140	GBP blood screen +	
		22; M		Blood 3200	-	
		40; M		Blood 40	-	
		24; F		Blood 50	-	
		29; M		Blood 120	-	
		21; M		Blood 80	-	
		24; M	Slurred speech, Lack of balance	Blood 76	BZD blood screen +	[115]
		22 *; Not reported	Moderate impairment	Blood 170	-	[86]
		42 *; Not reported	Mild impairment	Blood 12	-	
	Death	42; M	Accidental death complicated by obesity and asthma, Multiple drugs	Blood 386	MOR blood 116 COD blood 85, blood screen + HCOD urine screen +	[116]
		35; M	Multiple drugs	Blood 220	DZP blood 100 Nor-DZP blood 210 OXZ blood screen + TMZ blood screen + MTD blood 650, urine screen + EDDP blood screen + IBP blood screen +	[117]
		35; M		Blood 2520	EtOH blood 0.06 g/L BZD blood and urine screen + MOR blood 360, urine screen + COD blood 380, urine screen + PAR blood and urine screen +	
		Not reported	Multiple drugs	Blood 960	3OH-Penazepam blood 230 DZP blood screen + Nor-DZP blood screen + TMZ blood screen + MOR blood 10 MOR-3-glucuronide blood 30 MOR-6-glucuronide blood 10 PRZ blood 500	[118]
			Accidental overdose, Multiple drugs	Blood 960	3OH-Penazepam blood 270 DZP blood screen + Nor-DZP blood screen + DHC blood screen + DHC-6-glucuronide blood screen + NIC blood screen +	
		46; M	Phenazepam intoxication	Blood 1200	EtOH blood 0.22 g/L	[119]
		26; M		Blood 1600	DZP blood 160 DHC blood 160	
		Not reported	Multiple drugs	Blood screen +	MTD blood 60 MOR blood 60 COC blood screen + AMI blood 80 GBP blood 30,000	
				Blood screen +	Etizolam blood 34 EtOH blood 2.9 g/L MOR blood screen +	
				Blood screen +	DZP blood 240 MTD blood 890 MOR blood 30 DHC blood 170 GBP blood 26,000 MIR blood 100 FLX blood 140	
				Blood screen +	EtOH blood 3 g/L DZP blood 70 AMP blood 1500	
				Blood 10	MTD blood 770	
				Blood 140	EtOH blood 0.56	
				Blood 20	MTD blood 1300	
				Blood 20	EtOH blood 1.4 DZP blood screen +	
				Blood 24	Etizolam blood 120 MTD blood 950 COD blood 60 AMI blood 990	
				Blood 38	DHC blood 1100	
				Blood 40	MTD blood 700 MOR blood 50 AMI blood 570	
				Blood 40	MTD blood 340 DZP blood 350	
				Blood 40	MTD blood 390	
				Blood 43	DZP blood 510 MTD blood 900 MOR blood screen + MIR blood 580	
				Blood 45	Etizolam blood 73 DHC blood 300 GBP blood 42000	
				Blood 50	DZP blood 450 MOR blood 450	
				Blood 60	EtOH blood 0.79 g/L BUP blood 5 AMI blood 70	
				Blood 60	MTD blood 410	
				Blood 67	Etizolam blood 380 MOR blood 170	
				Blood 80	MTD blood 290	
				Blood 80	Etizolam blood screen + DZP blood screen + MOR blood 590	
				Blood 80	MTD blood 770 MOR blood 10	
				Blood 80	Etizolam blood screen + DZP blood screen + MOR blood 590	
				Blood 90	MOR blood 310	
				Blood 90	MOR blood 560	
				Blood 100	MTD blood 590 MOR blood 40	
				Blood 100	MTD blood 130 DOT blood 580	
				Blood 100	MTD blood 1200 BEG v blood screen +	
				Blood 100	MTD blood 280 DHC blood 1600	
				Blood 110	MTD blood 540 MOR blood 40 BEG blood screen +	
				Blood 110	OLZ blood 420 ZPC blood 10	
				Blood 110	EtOH blood 1.6 g/L BUP blood screen +	
				Blood 110	MTD blood 270	
				Blood 120	FEN blood 55 TMD blood 1400 DOT blood 3300 GBP blood 23,000	
				Blood 160	DZP blood 980 MOR blood 430 BEG blood screen +	
				Blood 200	MOR blood 360 DHC blood 990	
				Blood 210	MTD blood 180	
				Blood 240	MTD blood 390 MIR blood 60	
				Blood 240	DZP blood 350 MTD blood 340	
				Blood 240	MTD blood 510 AMI blood 840	
				Blood 260	MTD blood 240 COD blood 1100	
				Blood 280	EtOH blood 3.1 g/L	
				Blood 280	MTD blood 250 PGB blood 8000	
				Blood 330	MTD blood 750 MOR blood 330 GBP blood 103000	
				Blood 330	EtOH blood 2.5 g/L Nor-BUP blood 13	
				Blood 460	MTD blood screen +	
				Blood 550	EtOH blood 3.3 g/L	
				Blood 640	MTD blood 1100	
				Blood 820	MTD blood 470	
				Blood 900	DZP blood 120 MTD blood 380 DHC blood 730 AMP blood 110	
				Blood 1700	DHC blood 4400	
				Blood 1700	MOR blood 50	
				Blood screen +	DZP blood 170 TMD blood 7800 DHC blood 220	
Pyrazolam	Death	27; M	Multiple drugs	Blood 28 Central blood 28 Urine 500	Diclazepam blood 1, central blood 1, urine 1 DLP blood 100, central blood 250, urine 570 LMZ blood 6, central blood 4, urine 810 LZP blood 22, central blood 22, urine 820 3-FPM blood 10, central blood 9, urine 120 2F-MAMP urine 120 2F-AMP blood 89 MPA blood 2.2, urine 16 AMP blood 21, urine 75 DPH urine 340	[120]

^†^ Concentrations are expressed as ng/mL unless specified; + Positive; * Median age; 3F-AMP—3-Fluoroamphetamine; 3-FPM—3-Fluorophenmetrazine; 3,4-CTMP—3,4-dichloromethylphenidate; 4-ANPP—N-Phenethyl-4-piperidinone; 4-CIC—4-chloro-*N-*isopropylcathinone; 4-CMC—4-chloromethcathinone; 4F-MDMB-BINACA—Methyl 2-[1-(4-fuorobutyl)-1*H*-indazole-3-carboxamido]-3,3-dimethylbutanoate; 5F-AMB—*N*-[[1-(5-fluoropentyl)-1*H*-indazol-3-yl]carbonyl]-L-valine, methyl ester; 6-AM–6-Acetylmorphine; ALP—Alprazolam; AMI—Amitriptyline; AMP—Amphetamine; BE—Benzoylecgonine; BPP—Bupropion; BRP—Brorphine; BZD—Benzodiazepine; CBP—Cyclobenzaprine; CBZ Carbamazepine; CDP—Chlordiazepoxide; CE—Cocaethylene; CFN—Carfentanil; CIT—Citalopram; CLO—Clozapine; COC—Cocaine; COD—Codeine; CPA—Chlorpheniramine; CPZ—Chlorpromazine; CYC—Cyclizine; CZP—Clonazepam; DFSA—Drug-facilitated Sexual Assault; DIP—Diphenidine; DHC—Dihydrocodeine; DLP—Delorazepam; DOC—2,5-dimethoxy-4-chloroamphetamine; DOX—Doxylamine; DPH—Diphenhydramine; DOT—Dothiepin; DUID—Driving Under the Influence of Drug; DZP—Diazepam; ED—Emergency Department; EDDP—2-Ethylidene-1,5-dimethyl-3,3-diphenylpyrrolidine; EPH—Ephedrine; ESC—Escitalopram; EtOH—Ethanol; F—Female; FEN—Fentanyl; FLX—Fluoxetine; FZP—Flurazepam; GBP—Gabapentin; HCOD—Hydrocodone; HYZ—Hydroxyzine; IBP—Ibuprofen; ITZ—Isotonitazene; LEV—Levetiracetam; LMZ—Lormetazepam; LPM—Loperamide; LTG—Lamotrigine; LZP—Lorazepam; M—Male; MDA—Methylenedioxyamphetamine; MDMB-4en-PINACA—3-Methyl-*N*-[[1-(4-penten-1-yl)-1*H*-indazol-3-yl]carbonyl]-L-valine, methyl ester; MDMA—Methylenedioxymethamphetamine; MAMP—Methamphetamine; MDPV—3,4-Methylenedioxypyrovalerone; MDZ—Midazolam; MIR—Mirtazapine; MOR—Morphine; MPA—Methiopropamine; MPH—Methylphenidate; MTD—Methadone; MTG—Mitragynine; MXE—methoxetamine; NIC—Nicotine; MXP—Methoxphenidine; NTP—Nortriptyline; NZP—Nitrazepam; OLZ—Olanzapine; OD—Outpatient Department; OXY—Oxycodone; OXM—Oxymorphone; OXZ—Oxazepam; PAR—Paracetamol; PB—Phenobarbital; PEN—Pentedrone; PGB—Pregabalin; PMZ—Promethazine; PPL—Propranolol; PRO—Procyclidine; PRZ—Promazine; QTP—Quetiapine; RSP—Risperidone; SRT—Sertraline; TAP—Tapentadol; THC—Δ^9^-Tetrahydrocannabinol (Cannabis); THCCOOH—11-Nor-9-carboxy-THC; TMD—Tramadol; TMZ—Temazepam; TRZ—Trazodone; U-4770—*trans*-3,4-dichloro-*N*-[2-(dimethylamino)cyclohexyl]-*N*-methyl-benzamide; VEN—Venlafaxine; XYL—Xylazine; ZPC—Zopiclone.

## Data Availability

No new data were created or analyzed in this study. Data sharing is not applicable to this article.
